# Supplementary material for: Earth's magnetosphere and outer radiation belt under sub-Alfvénic solar wind
Source: Nat Commun. 2016 Oct 3;7:13001. doi: 10.1038/ncomms13001 (PMC5063966; doi:10.1038/ncomms13001)
Supplement: Supplementary Information — Supplementary Figures 1 – 10, Supplementary Notes 1 – 6 and Supplementary References [file ncomms13001-s1.pdf]

Supplementary Material

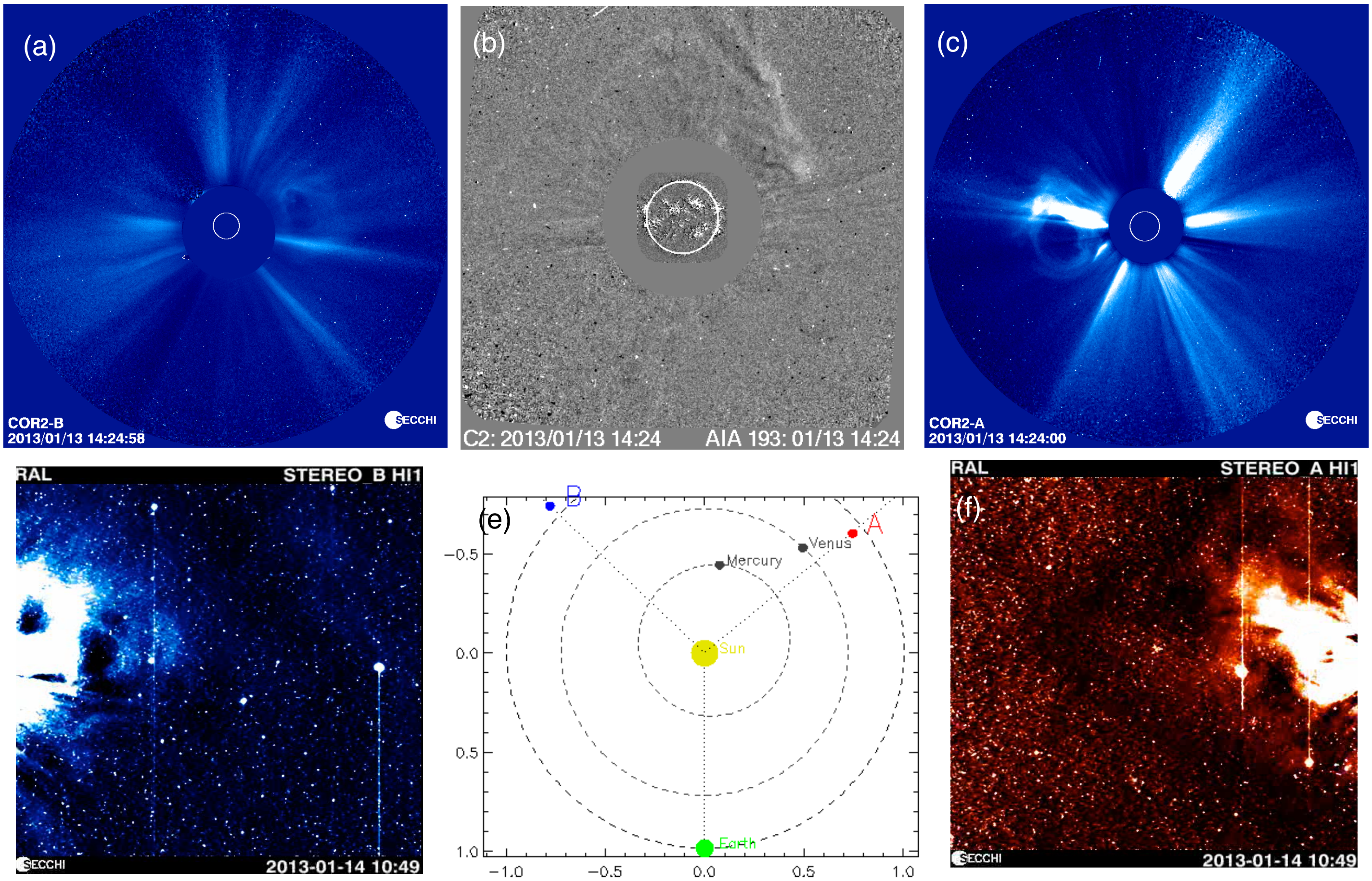

Supplementary Figure 1: White-light images of the CME which impacted Earth on 2013 January 17. Panels *(a), (b)* and *(c)* show SECCHI/COR2-B, LASCO/C2 and SECCHI/COR2-A images of the CME at 14:24 UT on January 13. The halo view from LASCO and limb view from STEREO-A and B confirm this is a Earth-directed event. Panels *(d)* and *(f)* show SECCHI/HI1 images of the CME at 10:49 UT on January 14. The STEREO spacecraft were each about 130° from the Sun-Earth line, as shown in panel *(e)*.

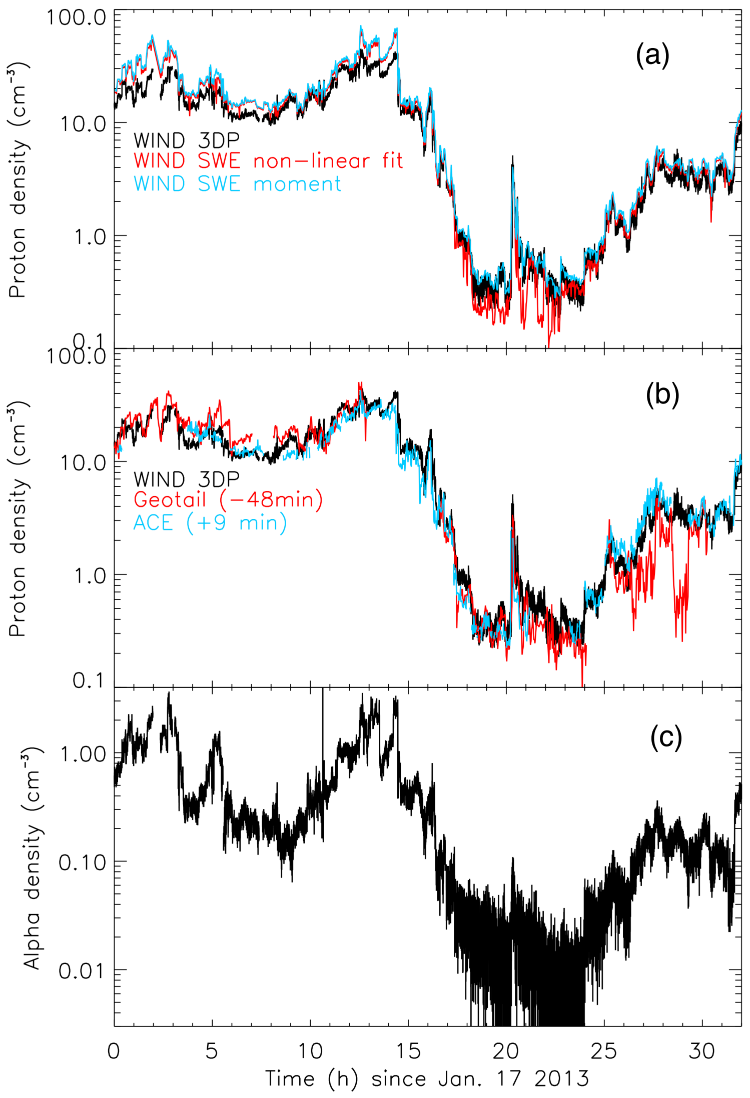

Supplementary Figure 2: Solar wind densities from different spacecraft, methods and/or instruments. Panel (a) shows the proton density as derived from Wind 3DP (black) and from Wind SWE through non-linear fit (red) and moment calculation (blue). Panel (b) shows the Wind 3DP proton density as compared to that measured by Geotail (shifted by 48 min) and by ACE (shifted by 9 minutes). Panel (c) shows the density of alpha particles as measured by Wind. These measurements validate the use of Wind 3DP in this study and confirm that from 17 to 24 UT on January 17 the solar wind density was below 1 cm^{-3}.

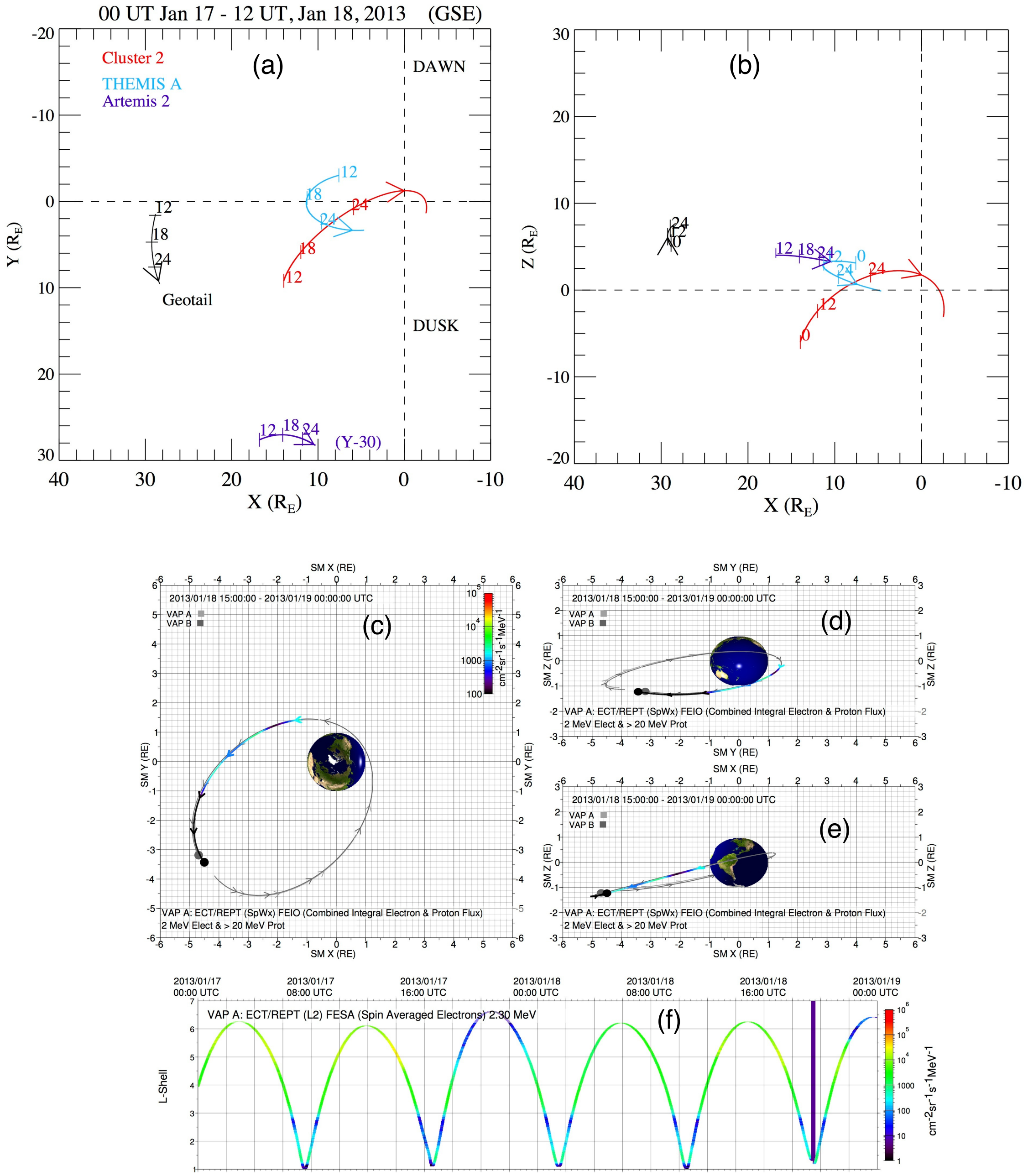

Supplementary Figure 3: Location of different spacecraft used in the study.
Panels (a) and (b) show the GSE coordinates (x-y and x-z, respectively) for Geotail, ARTEMIS-2, Cluster 2 and THEMIS-A. Panels (c), (d) and (e) show the SM coordinate for RBSP A and B in the x-y (panel c), y-z (panel d) and x-z (panel e) planes. Panel (f) shows the L-shell and flux of electrons at 2.3 MeV as measured by RBSP-A in the period of interest. The numbers in the panels (a) and (b) show the universal time (UT) in 01/17 at which a given spacecraft is at the location marked by the associated tick mark. The position of ARTEMIS-2 is plotted shifted by 30 R_E towards the Sun-Earth line (in the -Y direction). The main period of interest in the study is from 18:00 UT on January 17 to 02:00 UT on January 18, when all magnetosphere/solar wind spacecraft were at low Z values (in GSE coordinates).

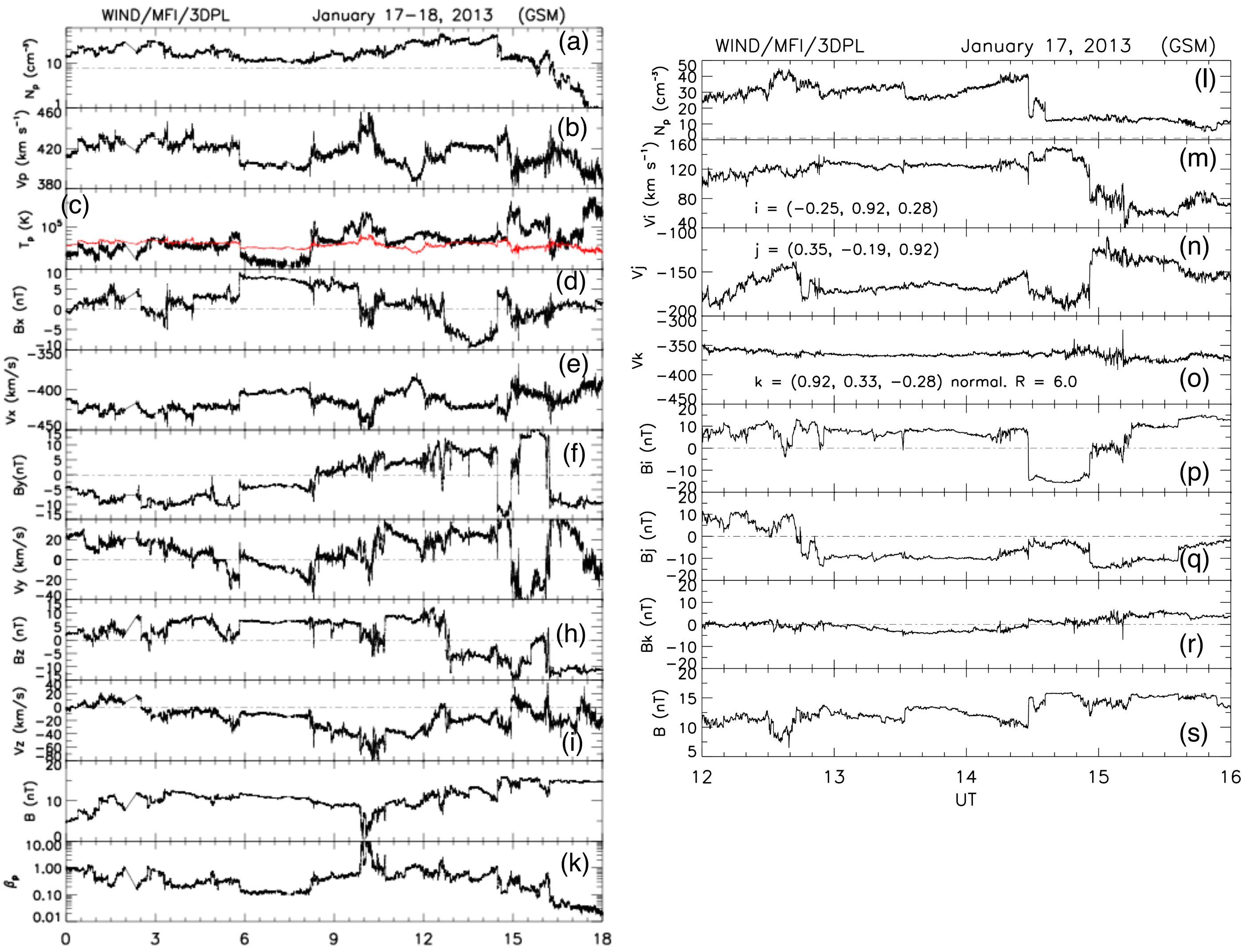

Supplementary Figure 4: Dense sheath before the magnetic ejecta on 2013 January 17. *Panels a—k:* Zoom-in on the sheath structure on January 17 from 00:00 UT to 18:00 UT as measured by Wind. The panels show the proton density (panel a), velocity (panel b), temperature (panel c), the x component of the magnetic field (panel d), the x component of the velocity (panel e), the y component of the magnetic field (panel f), the y component of the velocity (panel g), the z component of the magnetic field (panel h), the z component of the velocity (panel i), the total magnetic field strength (panel j) and the proton beta (panel k). The red line in the third panel shows the expected temperature following Lopez et al. (1987). *Panels l—s:* End of the sheath and beginning of the ejecta in the minimum variance coordinates. The panels show the proton density (panel l), the i, j and k components of the velocity (panels m—o), the i, j and k components of the magnetic field (panel p—r), and the total magnetic field strength (panel s).

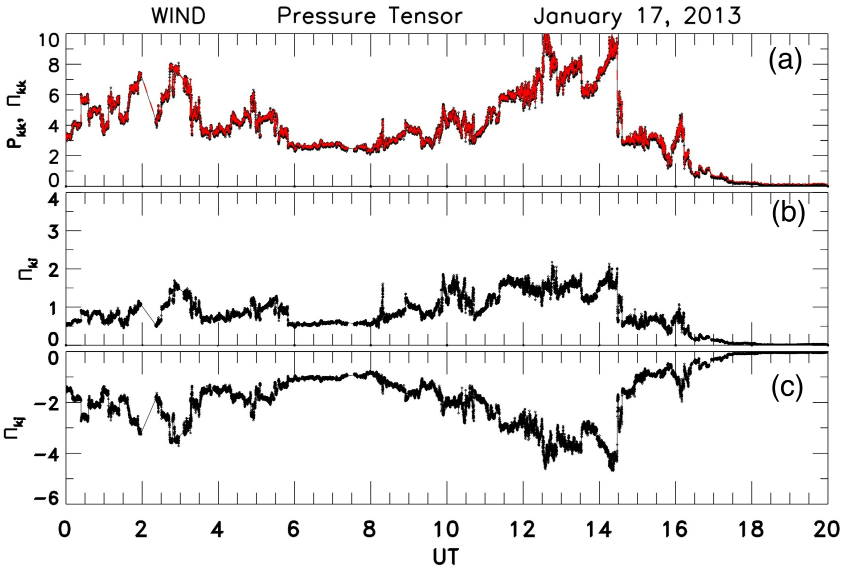

Supplementary Figure 5: Solar wind pressure tensor on January 17 as derived from Wind data. Panel (a) shows the total pressure term (black) and without the magnetic pressure (red) normal to the magnetopause. Panels b and c show the non-diagonal terms of the dynamic plus thermal pressure.

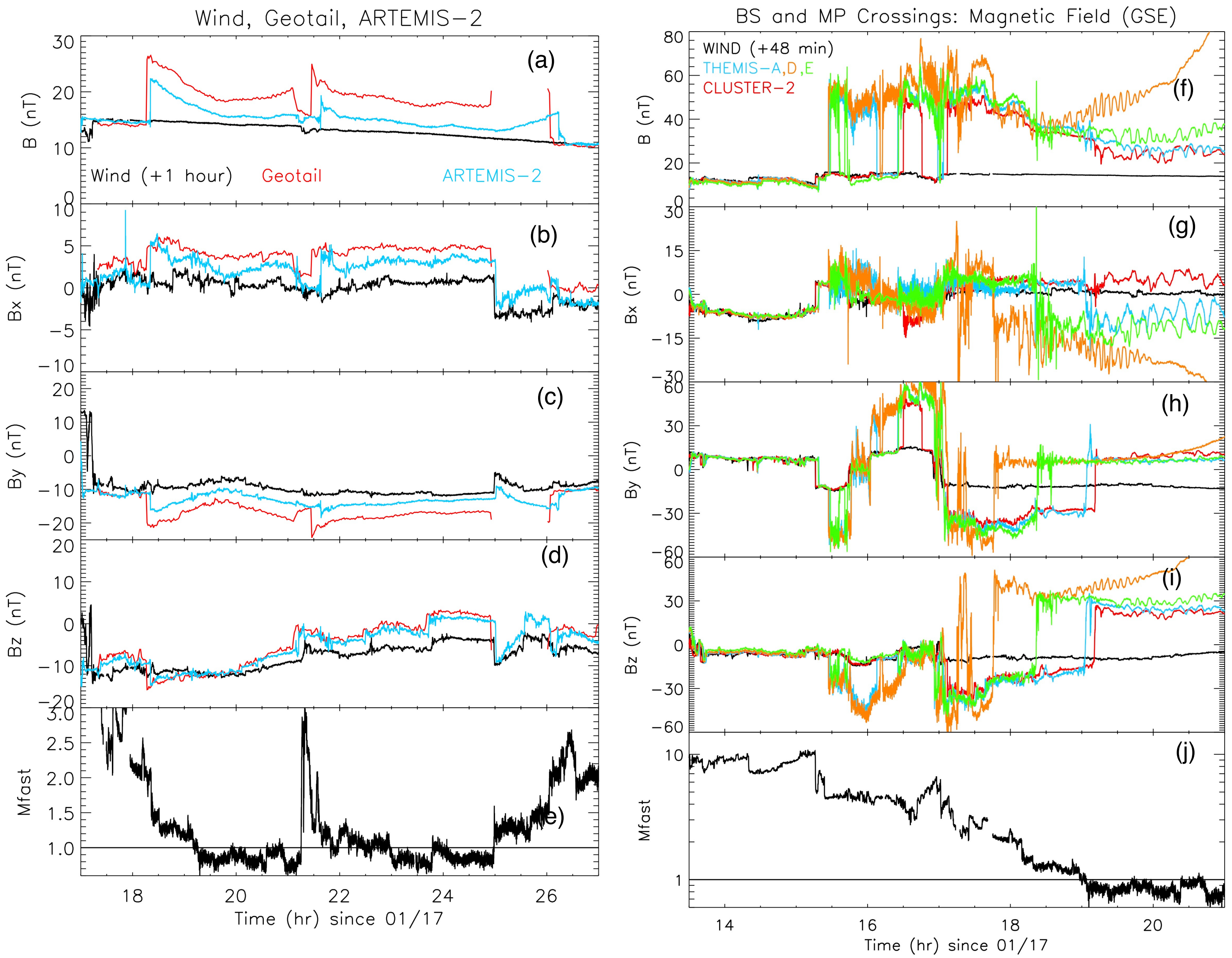

Supplementary Figure 6: Bow shock and magnetopause crossings.
Left: Geotail (red), ARTEMIS-P2 (blue) and Wind (black, shifted by 1 hour) measurements during January 17-18, 2013 showing the bow shock crossings, identifiable by large increase in the total magnetic field strength. Panels a and f show the total magnetic field strength, panels b—d and g—i the x, y and x components of the magnetic field and panels e and j the fast magnetosonic Mach number derived from Wind.
Right: THEMIS and Cluster-2 measurements showing bow shock crossings from 15:20 to 17:10 UT and magnetopause crossing from 17:40 to 19:10 UT and Wind time-shifted by 48 minutes. These crossings were used to create Figure~2.

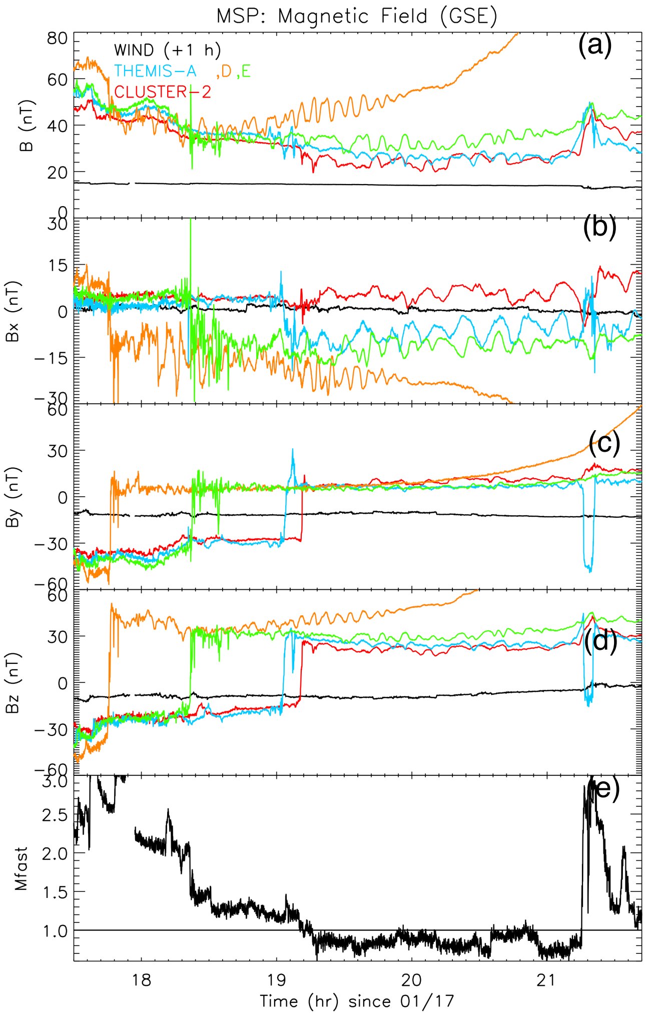

Supplementary Figure 7: Oscillations in the magnetic field as measured by THEMIS and Cluster-2. Panel a shows the total magnetic field strength, panels b—d the x, y and x components of the magnetic field and panel e the fast magnetosonic Mach number derived from Wind. Themis-A, D and E are plotted in blue, red and orange, respectively. Wind data is shifted forward by 1 hour and is plotted in black.

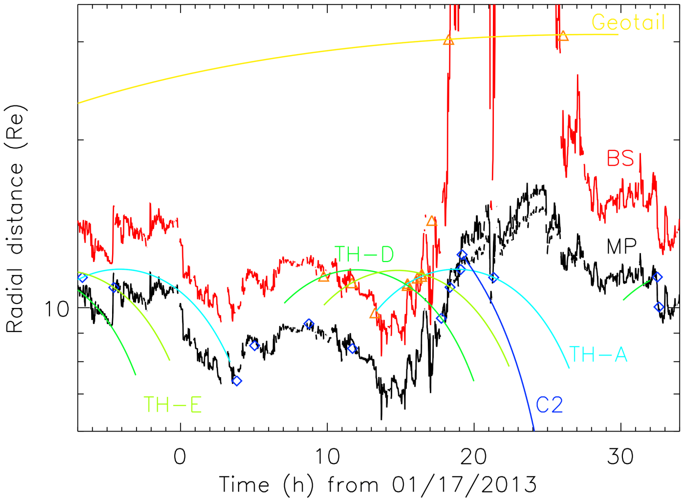

Supplementary Figure 8: Same as Figure 2 from the main article but with the magnetopause location following Shue *et al.* (1998) with the total pressure instead of the dynamic pressure in a light black line, which differ from the model of Shue *et al.* (1998) from hours 18 to 26.

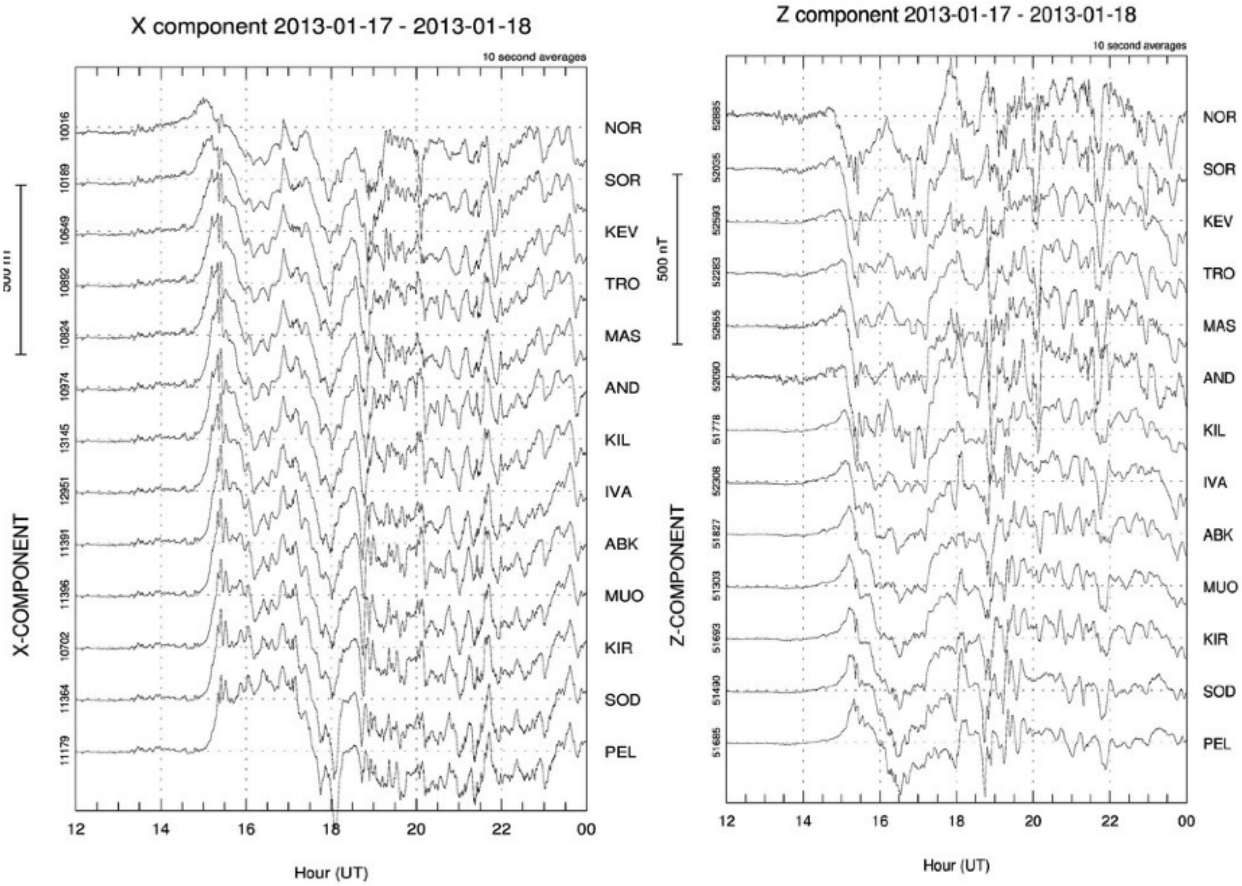

Supplementary Figure 9: X (left) and Z (right) components of the magnetic field measured by ground-based magnetograms part of the IMAGE chain from 12UT to 00UT on January 17 (Tanskanen, 2009). Clear perturbations can be seen starting around 15:00 UT, consistent with the arrival at the magnetopause of the first pressure drop. These illustrate how the magnetosphere was far from quiet at that time even during the sub-Alfvénic period which started around 19:00 UT and lasted until 01:00 UT on January 18, except for a 30-minute period around 21:15 UT.

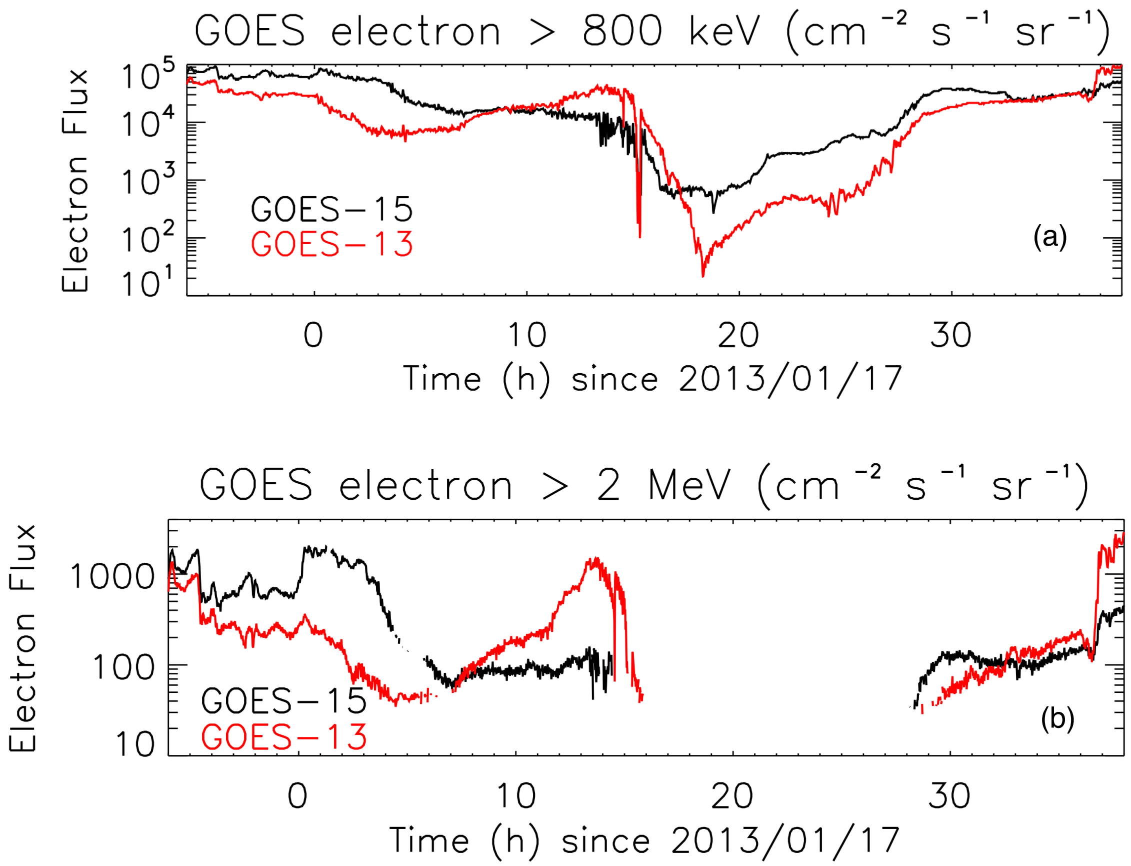

Supplementary Figure 10: Energetic electron measurements by GOES. GOES-13 (red) and GOES-15 (black) measurements of the flux of electrons with energies above 800 keV (panel a) and above 2 MeV (panel b) at geosynchronous orbit. The flux (y-axis) is measured in electron per cm^2 per s and per sr.

Supplementary Note 1- White-light observation of the CME:

The 2013 January 13 CME is observed by LASCO C2 and C3 coronagraphs (Brueckner *et al.*, 1995) as well as white-light imagers part of the SECCHI suite (Howard *et al.*, 2008) onboard STEREO, as shown in Supplementary Figure 1. In fact, the CME can be tracked using J-map (Davies *et al.*, 2009) until January 17 by STEREO-A/SECCHI and until January 15 by STEREO-B/SECCHI.

STEREO-B measurements analyzed with the self-similar expanding fitting technique (SSEF: Davies *et al.*, 2013) give a Earth-directed CME with a speed of ~470 km.s^{-1} (assuming a constant propagation speed) and a predicted arrival time on January 17 at 02:50 UT. This fitting result was obtained independently of the current study as part of a large survey undertaken under the HELCATS European project and further confirms that this CME is indeed the one which impacted Wind around 00:00 UT on January 17.
Although white-light images show a dark cavity (see for example top right panel of Supplementary Figure 1), the remote observations do not give any indication that the density inside the CME will be so low when the CME reaches 1 AU.

Supplementary Note 2- Solar Wind Density:

In the main text, we use the Wind spacecraft 3-D plasma analyzer (3DP: Lin *et al.*, 1995) analysis of the proton density to obtain the upstream solar wind proton density. In order to ascertain that instrumental effects are not the cause of the low-density period, we compare this density to that:

i) obtained from the Solar Wind Experiment (SWE: Ogilvie *et al.,* 1995) derived both through a non-linear fitting of the proton distribution function and from moment analysis.
ii) obtained from ACE Solar Wind Electron Proton Alpha Monitor (SWEPAM: McComas *et al.*, 1998)
iii) obtained from Geotail Comprehensive Plasma Instrumentation / Solar Wind Analyzed (CPI/SWA: Frank *et al.*, 1994).

In addition, we looked at the density of alpha particles obtained from Wind/3DP to make sure that the ratio of alpha to proton remains low.

Supplementary Figure 2 shows the comparison of the proton (and alpha for the last panel) density as measured by different spacecraft/methods, illustrating that all methods and instruments return a proton density below 0.4 cm^{-3} from 18:15UT to 00UT, except for the 30-minute period centered around 20:30UT, when the density was greater than 1 cm^{-3}. During the period of low density, the density of alpha particles is extremely low, reaching below 0.02 cm^{-3}. This shows that the alpha particles remain around the typical proportion of 5% (or 20% by mass) of the solar wind composition.
These comparisons validate the Wind/3DP density measurements and confirm that the period of interest was indeed sub-Alfvénic, and is not due to an instrumental failure or defect.

We further confirmed the low density by plotting (not shown here) the thermal noise receiver data from Wind (Meyer-Vernet *et al.,* 1998). It shows the plasma line dropping to value under 10 kHz around 17:30 UT, before a data gap. This is another, independent confirmation that the density of the solar wind was indeed very low at this time.

Supplementary Note 3- Planarity of the magnetic field in the CME sheath and pressure tensor

We performed a minimum variance analysis (MVA, Sonnerup and Cahill, 1967) on the magnetic field on January 17 from 00:00 UT to 16:00 UT. The ratio of intermediate to minimum eigenvalues is 7.8, indicating that the field is indeed planar. The normal to the plane is found to be (0.9183, 0.2315, -0.3210) in GSM coordinates, i.e. primarily in the Sun-Earth direction (*x*-direction).

In this coordinate system, B_n is equal to 0.6 ± 2.6 nT, much smaller than the other two components, which have magnitudes of the order of 10 nT, and consistent with $B_n \sim 0$ nT.
Overall, this planar structure is tangent to the magnetopause.

Supplementary Figure 4 shows a close-up to the sheath and the beginning of the ejecta, including the magnetic field in the MVA coordinate system.
There are strong correlation between non-radial components of the velocity (V_y and V_z) and of the magnetic field (B_y and B_z) especially during the late part of the sheath around 13:00UT. This further confirm the planarity of this structure.

Next, we determine, for the same period, corresponding to the dense sheath preceding the magnetic ejecta, the total pressure tensor, in order to identify stresses onto the magnetopause.

The total pressure tensor (total momentum flux tensor) is given by equation:

$$Pi_{\alpha\beta} = (P + B^2/(2\,\mu_0))\delta_{\alpha\beta} + \rho\, V_\alpha V_\beta + B_\alpha B_\beta/\mu_0, \qquad (1)$$

where α and β are running indices, ρ is the proton mass density, P the thermal pressure, B the magnetic field and V the velocity.
Using the coordinates i, j, k with $B_k = 0$, derived from the minimum variance of the field (see previous section), the component of momentum flux normal to the discontinuity plane is:

$$Pi_{k,k} = \rho\, V_k^2 + P + (B_i^2 + B_j^2)/(2\,\mu_0). \qquad (2)$$

Note that at the moment of impact, vector **k** is normal to both the discontinuity as well as to the magnetopause, so that $Pi_{k,k}$ represents the pressure normal to the boundary. During the first 13 hours of January 17, the dayside magnetopause at low latitudes can be modeled as a tangential discontinuity, since the field is northward (this excludes the cusp region). The components of the momentum flux in the magnetopause plane are given by: $P_{k,\beta} = \rho\, V_k V_\beta$, where β = i, j.
Supplementary Figure 5 shows the quantity $\Pi_{k,k}$ ($\equiv \rho\, V_k^2 + P$) and $P_{k,k}$ (red trace), followed by Π_{ki}, and Π_{kj}. It is clear from the first panel that the magnetic tension forces are negligible for these considerations.

Considering the changes in the pressure components, we see that (last panel) the $P_{k,i}$ is not negligible in comparison with the normal pressure in the top panel. So considerable tangential stresses were also applied to the magnetopause. These stresses dropped significantly around 14:30 UT (at Wind, corresponding to ~15:30 UT at the magnetopause) and became negligible around 16:30 UT.

Supplementary Note 4- Magnetopause and bow shock crossings by Geotail, THEMIS and Cluster and oscillations in the outer magnetosphere:

Earth's bow shock moved outward and crossed Geotail and ARTEMIS-P2 around 18:20 UT on January 17 (left panel of Supplementary Figure 6). During the period of low fast magnetosonic Mach number, ARTEMIS-P2 measures magnetic field within about 10% of what was measured far upstream by Wind (black), consistent with solar wind deflecting as it approaches Earth's magnetosphere. The bow shock crosses ARTEMIS-P2 once again, after the short period of super-fast flows around 21:20UT. Lastly, the bow shock crossed Geotail and ARTEMIS-P2 as it moved Earthward around 02:00UT on January 18.

As the THEMIS and Cluster spacecraft crossed the magnetopause into the dayside magnetosphere, they measured large oscillations in the magnetic field with amplitude of ± 5-10 nT lasting for several hours as shown in Supplementary Figure 7. These oscillations have varying periods of 5-10 minutes and appear to be associated with the large decrease in dynamic pressure occurring at the same time.

Supplementary Note 5- Magnetopause location taking into consideration the magnetic pressure:

We have used the model of Shue *et al.* (1998) to determine the subsolar distance of the magnetopause taking into consideration the influence of the dynamic pressure and of erosion when southward interplanetary magnetic fields are present. During the period of sub-fast and sub-Alfvénic flow, the dynamic pressure drops to about 0.1 nPa, which is comparable to the magnetic pressure. Since the proton beta is very low, the thermal pressure remains negligible as compared to the magnetic and dynamic pressures.

Although the model of Shue *et al.* (1998) was not designed to consider total pressure instead of dynamic pressure, the pressure balance at the magnetopause is certainly modified during the period of very low dynamic pressure. To test this effect, we have added replace the dynamic pressure in the formula of Shue *et al.* (1998) by the total pressure. This change makes a difference once the subsolar distance of the magnetopause reaches about 12 Re. The difference between neglecting the magnetic pressure or not is about 10% during this time. Supplementary Figure 8 is the same as Figure 2 from the main text but with the calculated magnetopause location using the total pressure in light black underneath the curve marked MP.

Supplementary Note 6 - GOES measurements

In addition to measurements by ECT (Spence *et al.,* 2013) and REPT (Baker *et al.,* 2013) onboard the Van Allen Probes, the response of Earth's outer radiation belts can be quantified using measurements of the flux of energetic electrons at geosynchronous orbit by the GOES satellite. Supplementary Figure 10 shows these measurements starting one day before the CME. With the arrival of the dense sheath and the rise in dynamic pressure at 00:00UT on January 17, there is a decrease of the flux of electrons of energies above 800 keV by a factor of 6-8. The main drop occurs around 15UT on January 17 with the drop in dynamic pressure (the solar wind

magnetic field turns southward earlier around 12 UT). The flux recovers early on January 18, nearly concomitantly with the end of the sub-Alfvénic period and the rise of the dynamic pressure.

Supplementary References:

Baker, D. *et al.,* The Relativistic Electron-Proton Telescope (REPT) Instrument on Board the Radiation Belt Storm Probes (RBSP) Spacecraft: Characterization of Earth's Radiation Belt High-Energy Particle Populations, *Space Science Reviews*, **179,** 337–381 (2013).

Brueckner, G. *et al*., The Large Angle Spectroscopic Coronagraph (LASCO), *Solar Physics*, **162**, 357-402 (1995).

Davies, J. *et al*., A synoptic view of solar transient evolution in the inner heliosphere using the Heliospheric Imagers on STEREO, *Geophys. Res. Lett.,* **36,** L02102 (2009).

Davies, J. *et al*., Establishing a Stereoscopic Technique for Determining the Kinematic Properties of Solar Wind Transients based on a Generalized Self-similarly Expanding Circular Geometry, *Astrophys. J.,* **777,** 167 (2013).

Frank, L. *et al*., The comprehensive plasma instrumentation (CPI) for the GEOTAIL spacecraft, *J. Geomagn. Geoelectr.,* **46**, 23-37 (1994).

Howard, R. *et al.,* Sun Earth Connection Coronal and Heliospheric Investigation (SECCHI), *Space Science Reviews*, **136,** 67–115 (2008).

Lin, R. *et al*., A Three-Dimensional Plasma and Energetic Particle Investigation for the Wind Spacecraft, *Space Science Reviews*, **71**, 125-153 (1995).

Lopez, R. E., Solar cycle invariance in solar wind proton temperature relationships, *J. Geophys. Res*., **92**, 11189-11194 (1987).

McComas, D. *et al*., Solar Wind Electron Proton Alpha Monitor (SWEPAM) for the Advanced Composition Explorer, *Space Science Reviews*, **86,** 563–612 (1998).

Meyer-Vernet, N. *et al.,* Measuring Plasma Parameters With Thermal Noise Spectroscopy, *Geophysical Monograph,* **103,** 205 (1998).

Ogilvie, K. *et al.,* SWE, A Comprehensive Plasma Instrument for the Wind Spacecraft, *Space Science Reviews*, **71**, 55-77 (1995).

Sonnerup and Cahill, Magnetopause Structure and Attitude from Explorer 12 Observations, *J. Geophys. Res.,* **72,** 171 (1967).

Spence, H. *et al.,* Science Goals and Overview of the Radiation Belt Storm Probes (RBSP) Energetic Particle, Composition, and Thermal Plasma (ECT) Suite on NASA's Van Allen Probes Mission, *Space Science Reviews*, **179,** 311–336 (2013).

Tanskanen, E.I., A comprehensive high-throughput analysis of substorms observed by IMAGE magnetometer network: Years 1993-2003 examined, *J. Geophys. Res.*, **114**, A0520 (2009).
